# Computational study of extrinsic factors affecting ACL strain during single-leg jump landing

**DOI:** 10.1186/s12891-024-07372-7

**Published:** 2024-04-23

**Authors:** Harish Rao, Ryan Bakker, Stewart McLachlin, Naveen Chandrashekar

**Affiliations:** Mechanical and Mechatronics Engineering, University of Waterloo, 200 University Avenue West, Waterloo, ON N2L 3G1 Canada

**Keywords:** Knee, ACL, Injury, Finite element, Jump-landing

## Abstract

**Background:**

Non-contact anterior cruciate ligament (ACL) injuries are a major concern in sport-related activities due to dynamic knee movements. There is a paucity of finite element (FE) studies that have accurately replicated the knee geometry, kinematics, and muscle forces during dynamic activities. The objective of this study was to develop and validate a knee FE model and use it to quantify the relationships between sagittal plane knee kinematics, kinetics and the resulting ACL strain.

**Methods:**

3D images of a cadaver knee specimen were segmented (bones, cartilage, and meniscus) and meshed to develop the FE model. Knee ligament insertion sites were defined in the FE model via experimental digitization of the specimen’s ligaments. The response of the model was validated against multiple physiological knee movements using published experimental data. Single-leg jump landing motions were then simulated on the validated model with muscle forces and kinematic inputs derived from motion capture and rigid body modelling of ten participants.

**Results:**

The maximum ACL strain measured with the model during jump landing was 3.5 ± 2.2%, comparable to published experimental results. Bivariate analysis showed no significant correlation between body weight, ground reaction force and sagittal plane parameters (such as joint flexion angles, joint moments, muscle forces, and joint velocity) and ACL strain. Multivariate regression analysis showed increasing trunk, hip and ankle flexion angles decreases ACL strain (R^2^ = 90.04%, *p* < 0.05).

**Conclusions:**

Soft landing decreases ACL strain and the relationship could be presented through an empirical equation. The model and the empirical relation developed in this study could be used to better predict ACL injury risk and prevention strategies during dynamic activities.

**Supplementary Information:**

The online version contains supplementary material available at 10.1186/s12891-024-07372-7.

## Background

Anterior cruciate ligament (ACL) injury rates continue to rise even with significant efforts directed towards understanding ACL injury mechanics and prevention strategies [1]. Close to 75% of ACL injuries in North America are non-contact in nature, occurring due to sudden dynamic movements in sport-related activities [2, 3]. Currently, there is a lack of consensus and validation of various biomechanical factors that lead to ACL injuries [4]. Both sagittal and frontal plane mechanics are known to contribute to ACL injury depending on the activity. Sagittal plane mechanics have been identified as one of the major factors contributing to ACL injury risk in drop-landing activities [4–7]. Quantifying the relationship between biomechanical factors, such as the critical knee kinematics [5–7], and the corresponding ACL strain during dynamic activities such as landing from a jump is challenging. There is a large body of research that measure loads and moments on the knee joint during landing [8–14]; however, it is difficult to translate such measurements and findings directly to the understanding of ACL strain. Direct in-vivo measurement of ACL deformation has been performed during activities of daily living such as squatting and stair climbing [15–18]. However, obtaining such measurements during dynamic activities, like a single leg jump landing that have a high potential for injury, is extremely difficult and often impossible. As an alternative approach, in-vitro experiments have been adopted to simulate dynamic knee loading conditions to understand the underlying joint mechanics [19–22]; yet, unless the dynamic loading conditions are painstakingly recreated, it is not possible to draw clear insights related to factors influencing ACL injury risk. A hybrid approach of an in-vivo motion capture study driving in-vitro cadaveric experiments with time-varying loading conditions of a jump landing was successfully explored in Bakker et al. (2016) [23]. It was found that the inherent knee anatomical features were major contributors to the resulting ACL strain compared to hip and knee kinematics. However, these hybrid experiments are complex and have small sample sizes due to the limited availability of cadaveric test specimens and the relatively few number of experiments that can be performed with each specimen. As such, it is not clear, if anatomic variability is controlled, what extrinsic factors affect ACL strain during activities such as jump-landing. This is important because extrinsic factors can be modified through training while intrinsic factors cannot be modified.

Computational modelling, such as finite element (FE) analysis, has been used extensively to study the behaviour of ACL [24–27]. A validated FE knee model with appropriate physiological kinematic and kinetic inputs can be effective in examining the relationship between various biomechanical factors and ACL strain during dynamic activities. Further, subject-specific FE models including accurate anatomical features can be powerful tools in predicting the individual risk of ACL injury [24, 28, 29]. There have been several recent studies that have used FE modeling for simulation of dynamic knee activities (such as jump landings) with appropriate physiological loading conditions in an attempt to better understand of ACL injury mechanics. For example, Yang et al. (2023) [25] created finite element models of thirty healthy subjects to study the stress and strain in the ACL at various flexion angles. Navacchia et al. (2019) [26] and Ueno et al. (2021) [27] both used validated FE models of knees to simulate dynamic activities using muscle forces and kinematics obtained from rigid body modelling of in-vivo motion capture. Using this approach, they were used to study the factors affecting the ACL strain. Their primary goal was to assess how knee abduction moment, anterior shear force, and internal rotation torque affect ACL force during landing. Hume et al. (2019) [30] developed a multi-scale FE model of the human lower extremity and combined optimization, muscle modeling, and FE analysis in a single pipeline to study the mechanics of healthy knees during dynamic activities. But they studied only non injurious activities such as chair raise and gait. None of the above studies, except the experimental study by Bakker et al. [23], presented an empirical model to quantify and predict ACL strain during dynamic activities. Such a model could potentially facilitate targeted athlete training programs aimed at quantitatively tracking progress in reducing ACL strain and associated injury risk.

The objectives of this study were to: (1) develop and validate a detailed knee FE model using published independent experimental data for multiple knee movements, (2) apply the FE model to simulate single-leg jump landings of ten participant kinetic and kinematic profiles to examine ACL strains, and lastly (3) quantify the influence of key extrinsic sagittal plane variables on overall ACL strain during jump-landing through an empirical model. This study builds on the study by Bakker et al. [23] and addresses the limitation related to anatomic variability in cadaver specimen by simulating multiple jump-landing scenarios on a single validated FE model of the knee.

## Methods

### Finite element model development

**Fig. 1:**
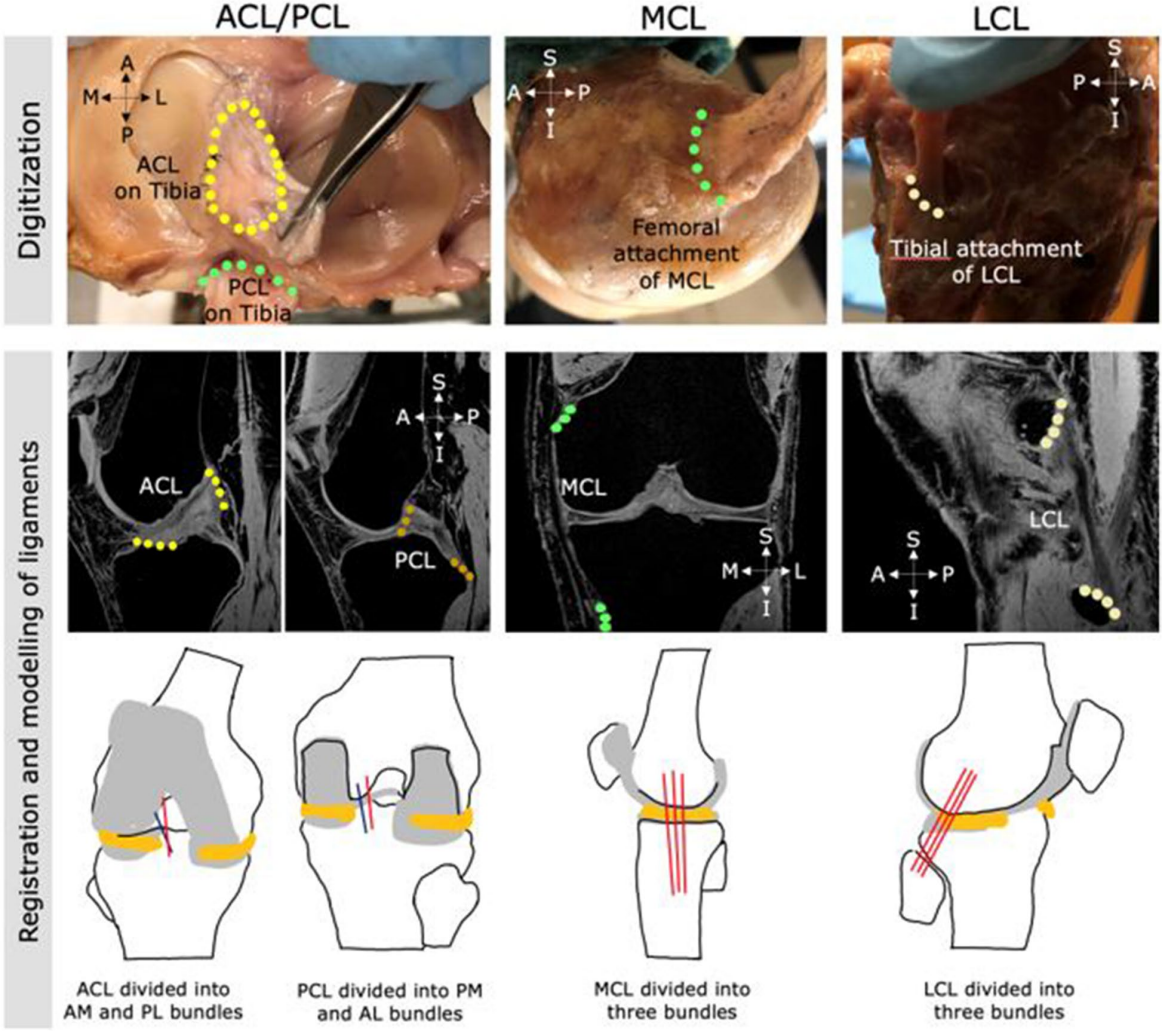
Digitization process of the ligament insertion sites of the ACL, PCL, MCL and LCL on the cadaver specimen (top). Registration of the acquired points in the MRI scans and construction of bundles ligaments (bottom)

Computed tomography (CT) and magnetic resonance imaging (MRI) of a fresh-frozen intact cadaver knee specimen (male, age: 49 years, mass: 77 kg, height: 178 cm) were obtained to develop a knee FE model. The specimen was healthy with no history of orthopaedic trauma or injury in the knee joint. The CT equipment used was Toshiba Aquilion CT scanner (Zoetermeer, NL) and the MRI equipment was Siemens MAGNETOM Prisma 3.0 T (Erlangen, Germany). On the MRI machine, the knee specimen was placed in a knee coil (Tx/Rx Knee 15 Flare Coil) to improve image quality. MRI was obtained in three commonly acquired sequences: T1-weighted, T2-weighted, and Proton Density (PD), for knee tissue segmentation [31]. The image resolutions were 0.415 × 0.415x0.833 mm for the CT and 0.3 × 0.3x0.5 mm for the MRI. The specimen contained the entire knee joint, including musculature and soft tissues. 3D Slicer (version 4.9) was used to define the hard and soft tissue geometries using a semi-automated 3D image segmentation technique [32]. Segmentations were defined for all bony tissues (femur, tibia, patella and the fibula), cartilaginous tissues (femoral, tibial and patellar cartilages) and the menisci geometries. The segmentations were clinically reviewed by an orthopaedic surgeon for accuracy. The cadaver knee was subsequently dissected and a MicroScribe-G2X™ (Immersion Corporation, USA) coordinate measuring device was used to digitize the ligament insertion areas of ACL, posterior cruciate ligament (PCL), lateral collateral ligament (LCL) and medial collateral ligament (MCL) on the cadaver specimen along with bony landmarks as detailed in Subburaj et al. (2009) [33] (Fig. 1).

The bones, including the femur, patella, tibia and fibula, were meshed with tetrahedral elements (C3D4). The femoral, patellar, lateral and medial tibial cartilages and the lateral and medial meniscus were meshed with (C3D8R) hexahedral elements. A MATLAB software library developed by Rodriguez-Vila et al. (2017) [34] was used to mesh the menisci, with the cartilages meshed separately using a block meshing approach with IA-FEMesh (MIMIX, The University of Iowa, IA, USA). The details of the mesh can be found in Table 1.

**Table 1:** Mesh details

Knee tissue	Type of mesh	No. of nodes	No. of elements	Average element edge length (mm)
Femur	Tetrahedral	38,442	190,673	2.75
Tibia		35,200	176,093	2.31
Patella		3630	17,096	2.20
Fibula		4804	21,037	2.10
Femoral cartilage	Hexahedral	13,924	9858	1.70
Lateral tibial cartilage		3880	2760	1.41
Medial tibial cartilage		3900	2772	1.34
Patellar cartilage		3596	2502	1.32
Lateral meniscus		2491	1840	1.23
Medial meniscus		1855	1360	2.07

**Fig. 2:**
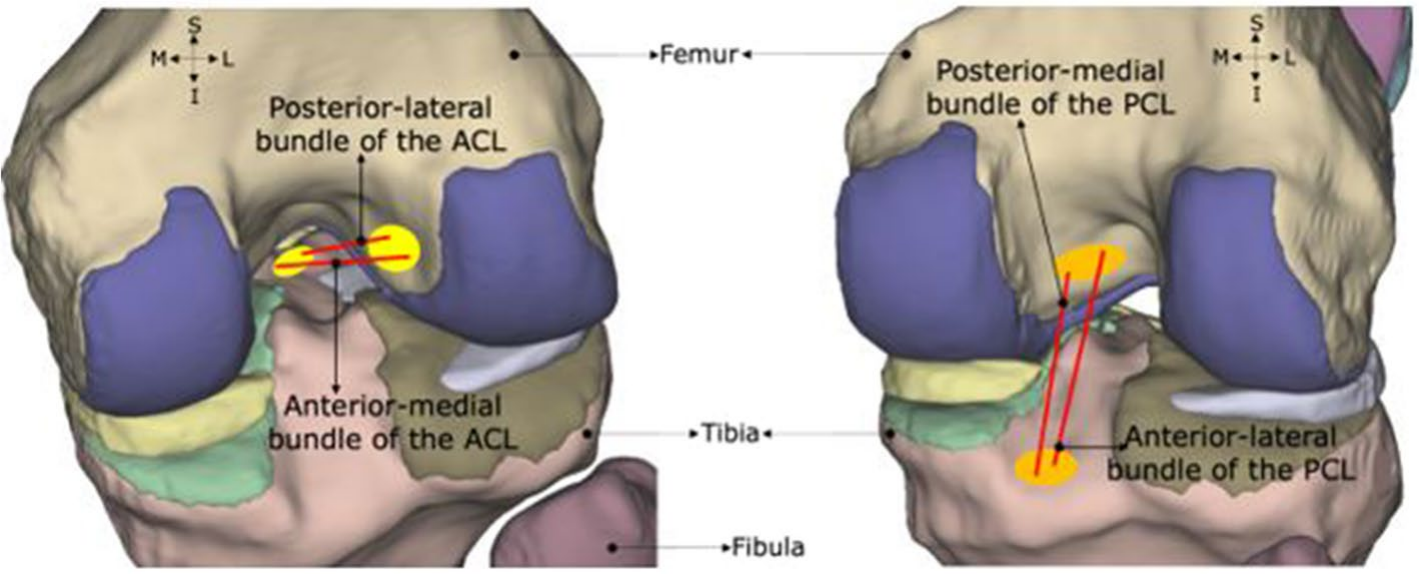
Knee model showing ligament insertion points of the ACL (left) and the PCL (right) bundles at their respective insertion sites, viewing the knee joint posteriorly

**Fig. 3:**
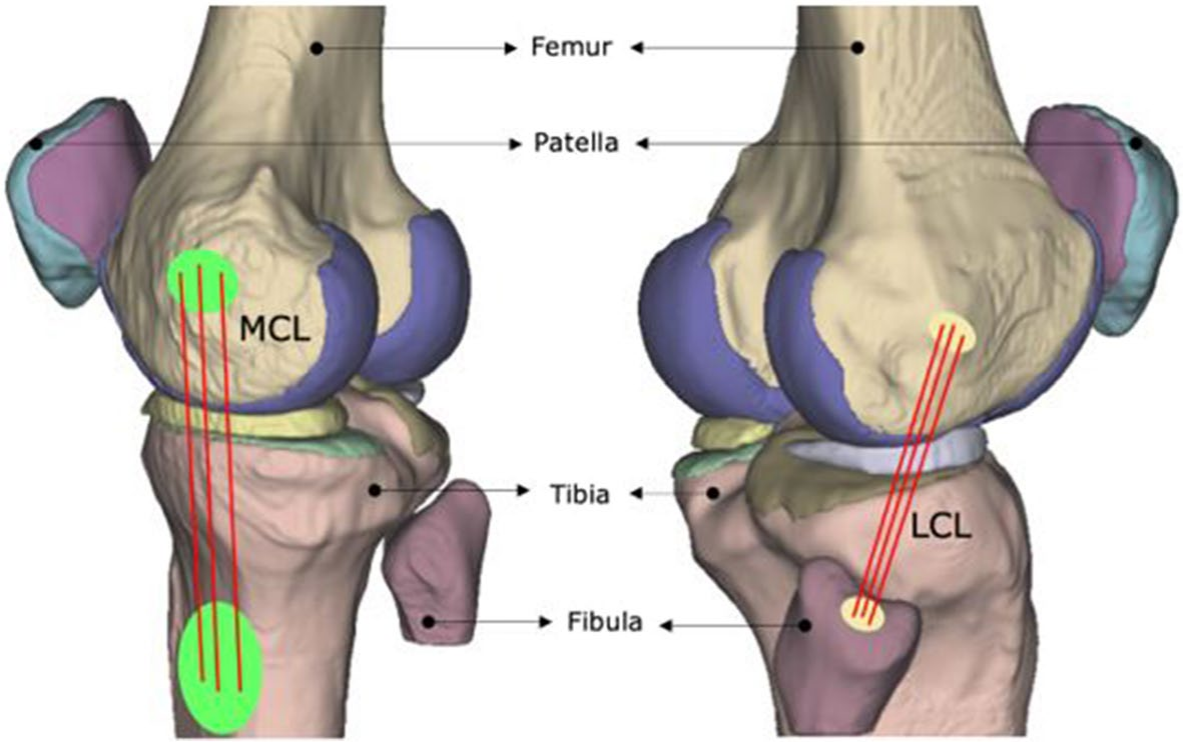
Knee model showing ligament insertion points of the MCL (left) and the LCL (right) bundles at their respective insertion sites

The meshed tissues (bones, cartilages, menisci) were imported to the finite element package Abaqus CAE 2018 (Dassault Systémes, Johnston, RI, USA). Linear elastic material properties were assigned to these tissues using values obtained from published literature data (see Additional file 1). Given the large disparity in stiffness between hard and soft tissues, the bones of the knee joint were modelled as rigid structures. The articular cartilage was assigned modulus of elasticity of 20 MPa and Poisson’s ratio of 0.45 [35], and the menisci were assigned modulus of elasticity of 59 MPa and Poisson’s ratio of 0.49 [36]. The ligaments of the knee joint were modelled as tension-only, non-linear axial spring elements (CONN3D2) in Abaqus based on the defined locations from the experimental digitization of the cadaver specimen (Figs. 2 and 3). The ACL and PCL were each modelled with two bundles; the MCL and LCL with three bundles each (Figs. 2 and  3). Non-linear force versus displacement relationship was assigned to the ligamentous structures according to the mathematical model developed by Blankevoort et al. (1991) [37]. The material properties of ACL were obtained from an experimental study Chandrashekar et al. (2006) [38]. The stiffness and reference strain parameters applied to each 1D element representing the ligaments is presented as Supplementary Material data (see Additional file 1).

**Fig. 4:**
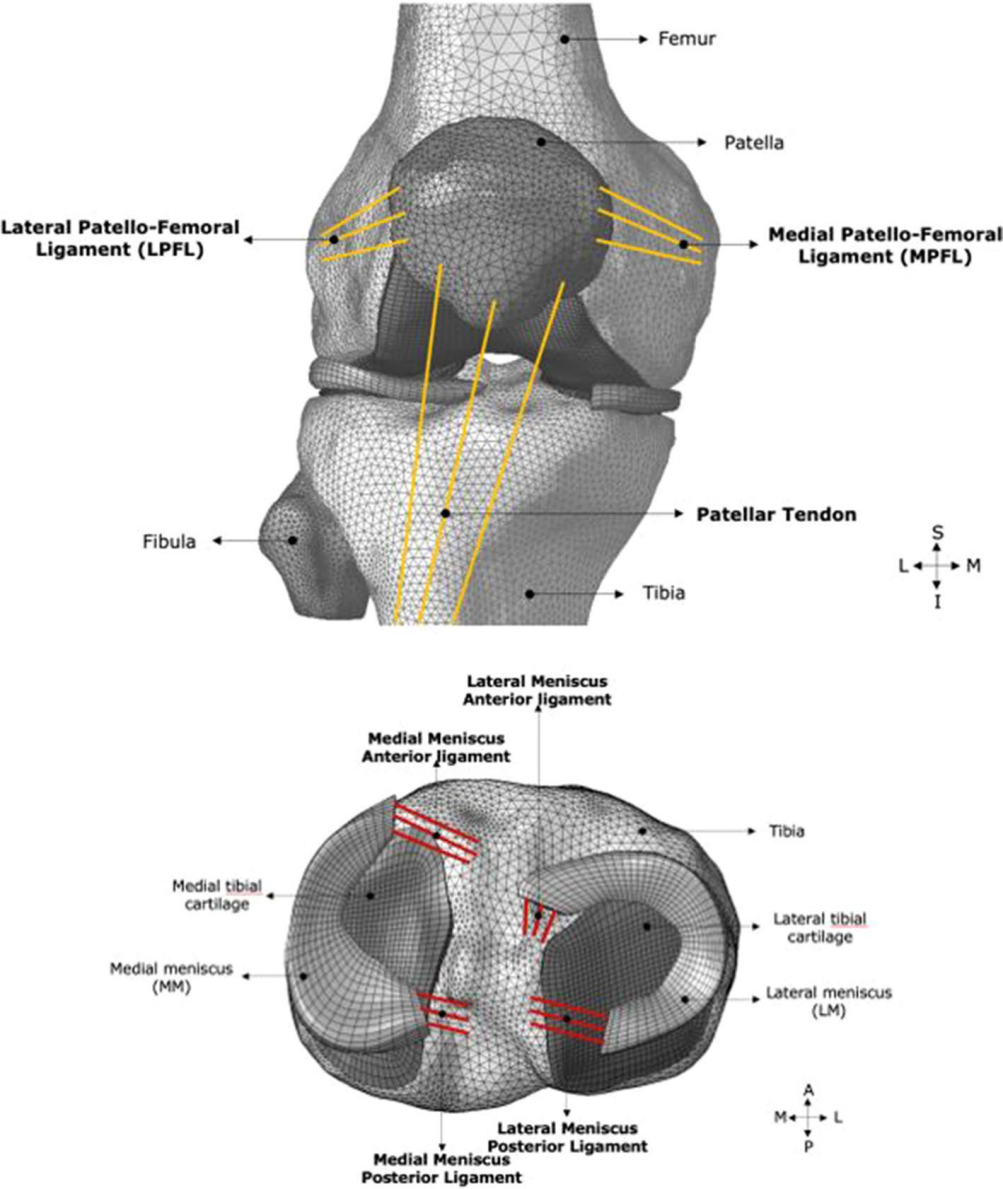
Knee FE model showing patellar (top) and meniscal (bottom) ligament insertion points at their respective insertion sites

In addition, other ligaments such as the patellar ligament (PL, k = 545 N/mm) [39], medial and lateral patellofemoral ligaments (MPFL, k = 16 N/mm and LPFL, k = 12 N/mm) [40] were included in the model and their attachment sites were obtained from published data [41]. The menisci were attached to the tibia via the meniscal horn attachments (k = 180 N/mm) [42]. All contacts were defined as frictionless. The assembled FE model with ligaments modelled as connector elements is shown in Fig. 4.

### Finite element model validation

**Fig. 5:**
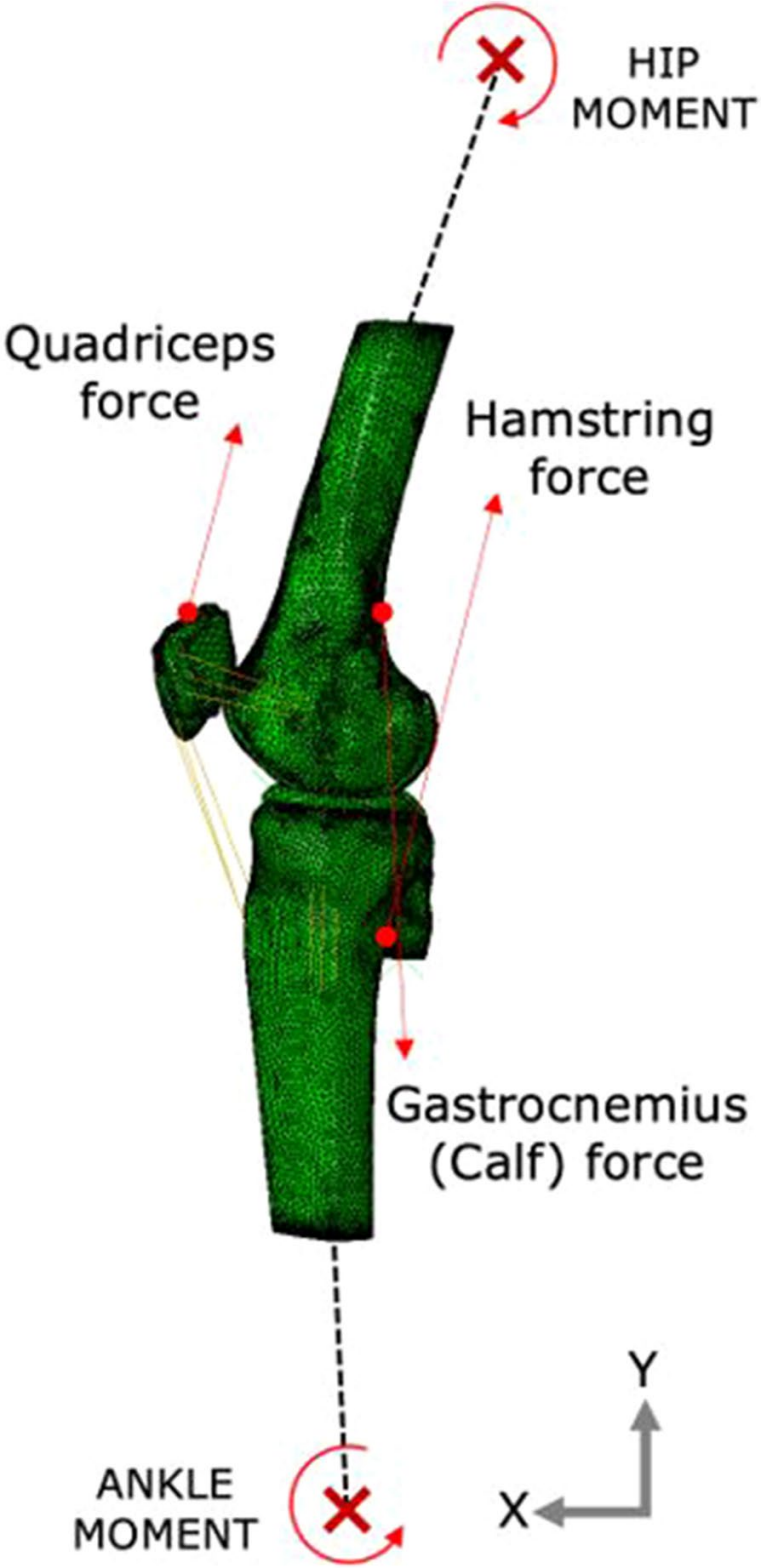
Location of application of muscle forces and kinematics in the FE model

The models’ ACL strain response to kinematic and kinetic stimuli was separately validated against published basic knee movements – knee flexion, Lachman test, anterior drawer test (in the sagittal plane), abduction (in the frontal plane) and internal rotations of the tibia during flexion (in the transverse plane). For each motion, model kinematics were compared to the published results and no adjustments were made to the material properties to better match the results. The boundary conditions were a combination of muscle forces and kinematic constraints at the locations as shown in Fig. 5. Flexion was simulated by applying a hamstring force of 600 N and constraining the femur. The resulting ACL strain and internal rotation of the tibia during flexion were compared to two in-vivo studies: Beynnon et al. (1992) [43] and Kiapour et al. (2013) [24]. In the coronal plane, abduction was simulated by applying up to 50 Nm abduction moment to the tibia. ACL strain during abduction and knee valgus rotation was compared to the experimental study of Kiapour et al. [24]. Lachman and anterior drawer tests were simulated at 30° and 90° flexion angles with anterior tibial force of up to 200 N and the resulting ACL strain was compared to the in-vivo studies of Beynnon et al. [43].

### Simulation of single-leg jump landing

**Fig. 6:**
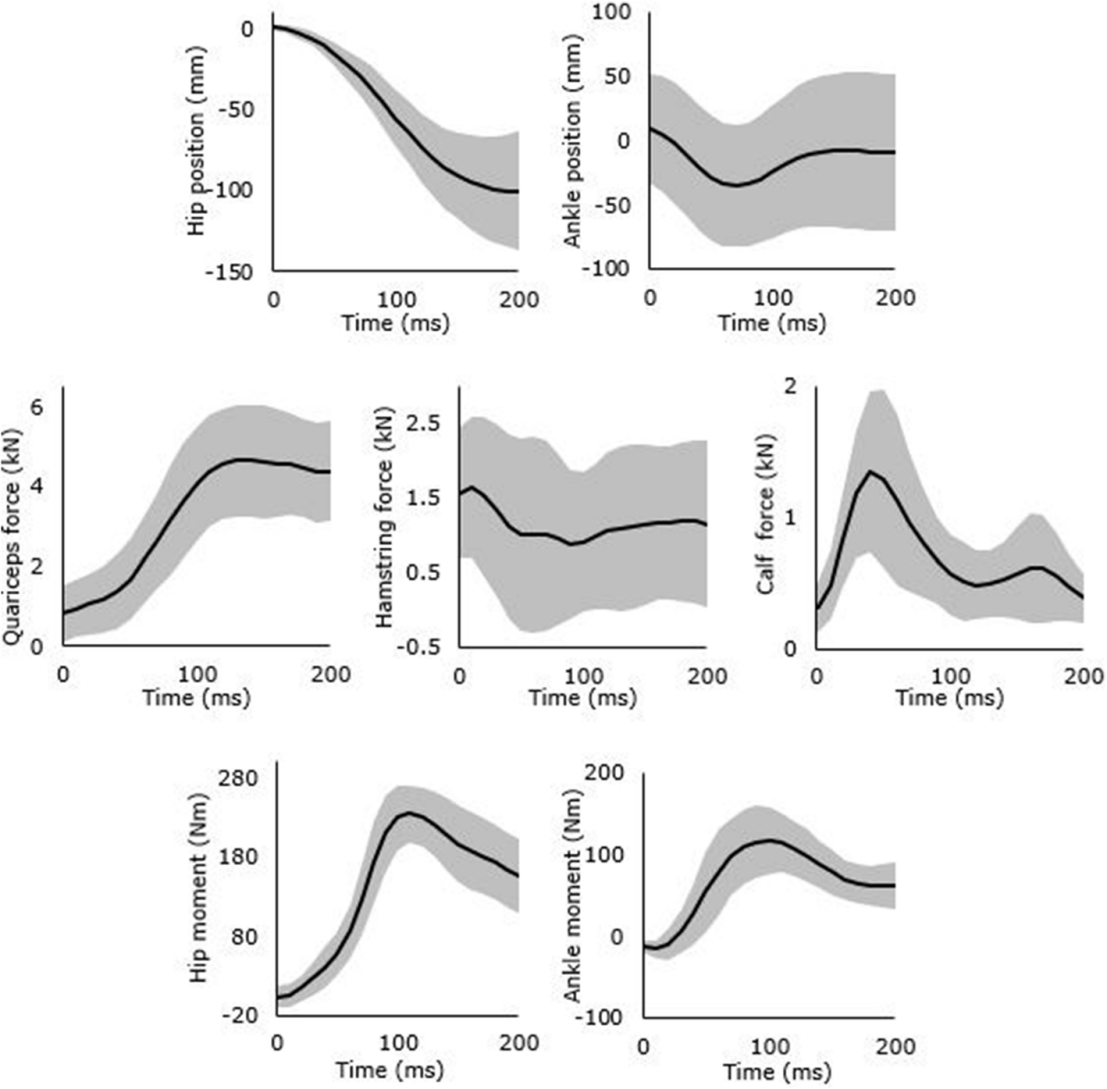
Input boundary conditions of single-leg jump landing, showing the average (black) and ± 1 standard deviation (gray) of Hip and Ankle position (row 1), Quadriceps, Hamstrings and Calf Muscle Forces (row 2) and Hip and Ankle Moments (row 3)

Bakker et al. [23] performed motion capture on 10 participants performing single-leg jump landing and obtained dynamic muscle force–time curves and kinematics of the hip and ankle using OpenSIM software [44]. The anthropometric parameters of these 10 participants is presented in supplementary data (see Additional file 1). These ten jump landing kinematic and muscle forces profiles from Bakker et al. [23] were simulated on the knee FE model using Abaqus Explicit dynamic analysis. The boundary conditions applied to the model were quadriceps, hamstrings and gastrocnemius muscle force profiles, hip and ankle moments and displacements. The average and standard deviations of the kinetics, kinematics, and the dynamic muscle force profiles, which are the boundary conditions to the FE model are shown in Fig. 6. The relative strain in the ACL was calculated using the length of the ACL bundles during touch-down as gauge length. The results were then compared to the increase in strain during landing phase to the results found in Bakker et al. [23].

### Statistical analysis

To compare the ACL strain between different participants, the peak ACL strains were normalized by subtracting the mean and dividing by the standard deviation. A bivariate Pearson correlation analysis was performed on critical kinetic and kinematic variables in the sagittal plane such as joint peak flexion angles, peak joint moments, peak muscle forces, and joint velocity obtained from motion capture and rigid body simulation [23] (independent variables) with normalized peak ACL strains obtained from the FE jump landing simulations (dependant variable). A multivariate regression analysis was performed to develop an empirical model to predict peak ACL strain during jump landing using the sagittal plane parameters thereby quantifying the biomechanical parameters that affect ACL strain. Box-Cox transformed strain data was used to generate the empirical models, with alpha = 0.05 in Minitab statistical analysis software (Minitab, Pennsylvania, USA). Box–Cox transformed strain was needed to stabilize the variance between the profiles while still including a constant factor in the regression analysis.

## Results

### Finite element mesh quality results

The mesh quality of all hexahedral elements was assessed in HyperMesh (Altair, Michigan, USA). A Jacobian value greater than 0.6 was observed in more than 97% of all elements, and warpage less than 15° was found in more than 99.7% of the elements. An aspect ratio of less than three was identified in more than 70.6% of the menisci elements. In the meshes of all other structures, aspect ratios of less than three were observed in more than 83% of the elements. Furthermore, internal and external angles in the range of 45°—135° were present in more than 88.5% of all elements, except for the medial meniscus, where only 65.2% of the elements fell within the preferred angle range. These meshes were deemed to meet the criteria suggested by Yang (2018) for acceptable quality [45].

### Knee motion validation of the finite element model

**Fig. 7:**
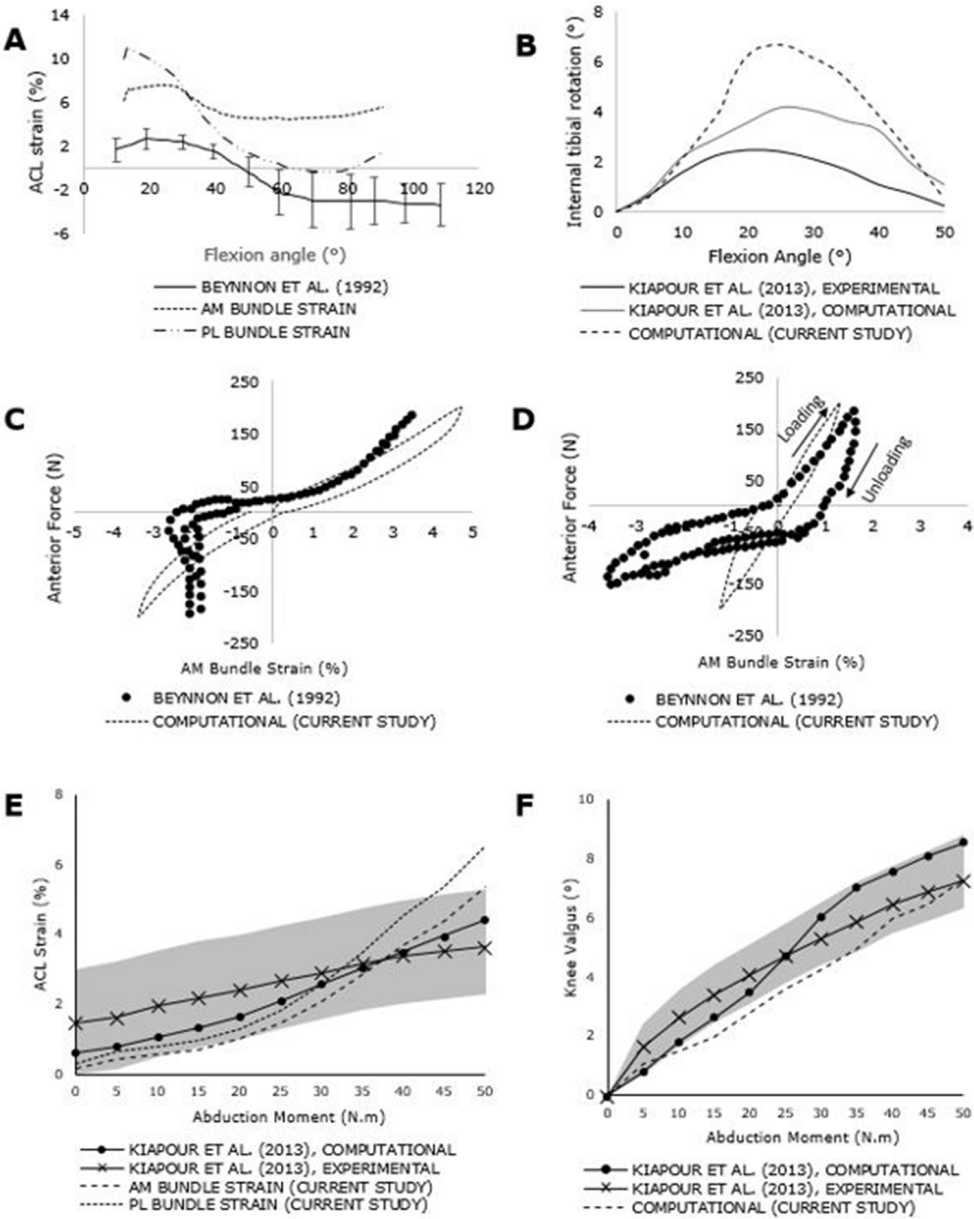
Independent methods of computational model validation. **A** ACL strain with increasing flexion angle (active range of motion). **B** Internal tibial rotation with flexion (**C**) AM bundle strain during Lachman test (**D**) AM bundle strain during anterior drawer test (**E**) ACL strain during knee abduction (**F**) Knee valgus rotation during knee abduction. The shaded area represents experimental 95% confidence intervals from Kiapour et al. (2013)

Kinematic validation of the FE model with respect to ACL strain was conducted, encompassing coronal, axial, and sagittal plane mechanics, and compared against published data. In Fig. 7A, the strain pattern of the AM and the PL bundles during flexion is depicted and compared to in-vivo experimental values from Beynnon et al. [39]. The validation of the internal rotation of the tibia ("screw home mechanism") [42] is presented in Fig. 7B, referencing the in-vitro experimental and computational study by Kiapour et al. [24]. Sagittal plane motion was validated using Lachman and anterior drawer tests. ACL strain during ± 200 N anterior–posterior loads on the tibia was compared to the in-vivo data of Beynnon et al. [39], as illustrated in Figs. 7C and D. Coronal plane motion was validated through the simulation of pure abduction motion, involving a 50 Nm abduction moment on the tibia. Figure 7E showcases the comparison of computational ACL strain with the in-vitro and computational results of Kiapour et al. [24], while Fig. 7F demonstrates knee valgus rotation with increasing abduction moment. To compare the computational results of the current study on model validation with published experimental results, a correlation analysis was performed (Table 2). For most cases, a high level of agreement was observed between the current model and published results, with a correlation coefficient (r) greater than 0.9.

**Table 2:** Correlations with experimental data of validation studies

Loading condition	Validation parameters	Model vs. Experimental data		Reference study
		Pearson correlation (r)	RMSE	
**Up to 50 N.m abduction moment at 25° flexion**	Knee valgus AM bundle strain PL bundle strain	0.98 0.94 0.94	0.89**°** 1.2% 1.3%	Kiapour et. al (2013) [24]
**Flexion up to 90°**	AM bundle strain Int. rotation of the tibia	0.82 0.90	6.2% 2.6**°**	Beynnon et. al (1992) [43] Kiapour et. al (2013) [24]
**Lachman test (at 30° flexion)**	Anterior loading	0.97	0.57%	Beynnon et. al (1992) [43]
**Anterior draw test (at 90° flexion)**	Anterior loading	0.98	0.28%	Beynnon et. al (1992) [43]

### Simulation results from single-leg jump landing

**Fig. 8:**
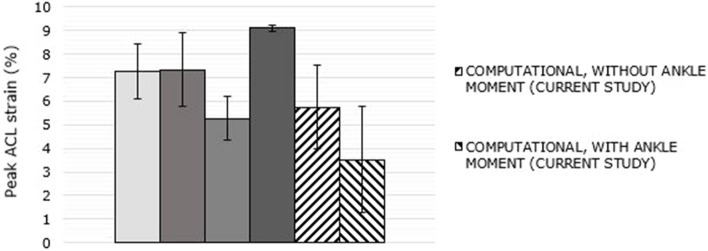
Comparison of peak ACL strains of P1-P7 profile jump landings with Bakker et al. (2016). Each of the 4 shaded bars represent strains obtained from in-vitro experiments simulating jump-landing of 7 participant profiles. Hatched bars represent the current computational study

The peak ACL strain values from the current study were compared to the ACL strains measured by Bakker et al. [23], who employed the same 10 muscle force and kinematic profiles. It should be noted that the ankle moment was not applied in their experiments due to limitations in the experimental setup. This comparison is visually presented in Fig. 8. Among the five specimens tested by Bakker et al.23, 'Knee 1' exhibited significantly higher strains, reaching peak strains that were 3x-5 × greater than those of the other specimens. Consequently, 'Knee 1' was excluded from the statistical comparison. The peak ACL strain, with and without the application of the ankle moment, was found to be 3.5% ± 2.2% and 5.4% ± 2.6%, respectively, indicating a 35% reduction in average ACL strain when the ankle moment was introduced.

The results of the bivariate analysis (based on Pearson correlation test) of the critical kinetic and kinematic sagittal plane parameters from motion capture and OpenSIM simulations and the resulting maximum ACL strain from the corresponding FE simulations are provided in Supplementary Data (refer to Additional file 1). While several sagittal plane parameters exhibited moderate correlations (Pearson correlation coefficient > 0.6) with maximum ACL strain, none of them were deemed significant contributors to ACL strain (*p* < 0.05).

A multivariate regression equation was formulated to predict peak ACL strain by incorporating independent variables. It was observed that each parameter or independent variable incorporated into the regression model made a significant contribution to the R^2^ value (*p* < 0.05). The model itself exhibited an R2 value of 90.04%. Equation 1 presents the multivariate regression model, while Table 3 presents the individual contributions.

**Table 3:** Components of peak ACL strain regression equation

Source	Co-efficient	*p*-value	Contribution
Regression		0.01	90.04%
Trunk flexion @ max. GRF	-0.1649	0.002	43.43%
Max. Knee flexion	0.0701	0.011	31.55%
Ankle flexion @ max. GRF	-0.2661	0.003	8.44%
Hip flexion @ max. GRF	-0.1362	0.002	6.62%
Constant term	9.09	0.001	
Error			9.96%
Total			100%

1 $${\text{ln}}\left(peak\, ACL\, strain\right)=\ 9.09+0.07\left(max. knee\, flexion\right)-0.266\left(ankle\, flexion\, at\, max. GRF\right)-0.136\left(hip\, flexion\, at \,max. GRF\right)-0.165\left(trunk\, flexion\, at\, max. GRF\right)$$

## Discussion

In the current study, a FE model of the knee joint was developed to simulate a dynamic loading scenario of single-leg jump landing with the goal of isolating the extrinsic factors affecting ACL strain. This is one of only a few reported mathematical [40, 41] and FE models [24, 46, 47] developed specifically to investigate jump landing motion involving dynamically varying muscle forces with hip and ankle flexion moments. The empirical model to predict ACL strain presented in this study is one of the only two such models available.

The current study used the same knee anatomy and material properties for all 10 kinematic profiles to isolate the effect of extrinsic factors on ACL mechanics while intrinsic variations were kept constant. This type of approach would not be possible with an experimental cadaver study. Our corresponding experimental studies have found it infeasible to reliably apply the 10 different kinematic profiles on a single cadaver knee as the knee tissues are likely to fail when repetitious, aggressive muscle force profiles are applied in an experimental setup. This would necessitate multiple specimens being required for different kinematic profiles, which would result in undesired variation in the measured data as a result of intrinsic factors, such as the tissue geometry and material properties.

Strengths of the current model include high-quality meshes for soft tissues to minimize numerical errors for dynamic analysis and accurate locations of ligament insertion sites that are directly measured from the dissected cadaver knee rather than through imaging. This approach addresses the limitations of in-vivo and in-vitro experimental studies and incorporates physiological loading conditions in the simulation of a dynamic activity.

### FE model and validation

The mesh quality used in this model was very high [45]. However, due to the inherently curved structure of the menisci, the aspect ratio and internal angle requirements were met only by 65% of the meniscal elements. Yang [45] states that the complete elimination of all elements with low-quality would require considerable time and effort, and hence, it is generally acceptable to have a small percentage of sub-par elements in an FE model of biological tissues with high-curvature.

Realistically, all knee soft tissues exhibit anisotropic, non-linear behaviour under loading. A few computational studies have implemented complex anisotropic hyperelastic models including poroviscoelastic features to describe the behaviour of cartilage and menisci [48–50]. However, in many computational models of the knee joint, where the focus of the study was on the ligament mechanics, linearly/transversely isotropic material properties were used [24, 51]. Similarly, in this study, isotropic, linear elastic material properties were assigned to the cartilages and menisci, with values obtained from published literature.

In this study, the ligaments were represented as 1D non-linear spring elements, which required only the location of their insertion on the bones. Despite the availability of complex models to describe ligament behaviour in a FE model, Beidokhti et al. (2017) [52] evaluated 1D and 3D continuum models of ACL and concluded that 1D models produce quick and satisfactory results if the kinematic output from the simulation is the main objective of a study. Several computational FE models have optimized ligament properties to match experimental simulations [25, 26]. However, The primary focus of our study was to investigate the impact of sagittal plane parameters on ACL strain while keeping other intrinsic parameters constant. For this purpose, having reasonably accurate geometry and mechanical properties was considered sufficient. Therefore, no efforts were made to optimize the material properties to exactly match the experimental studies unlike the studies by Beidokhti et al [53]., and Harris et al [54]. Rather, the model's validation aimed to ensure its reliability by comparing simulation results with published data, similar to the approach used by Bloemker et al [55]. and Zielinska et al [56]. Although the match is not perfect, the trends and order of magnitude support the model's performance in achieving the study's objectives. Therefore, the mathematical model detailed in Blankevoort et al. [37] was used as the source of force versus displacement curves for the 1D ligaments, including ligament pre-strains.

The kinematics of the knee FE model were validated against published literature data under quasi-static and dynamic loading conditions. Basic knee motion such as flexion/extension and pure abduction were simulated, along with Lachman and anterior draw tests which are the common physiological tests performed on a patient to identify ACL tears. During flexion, AM bundle strain decreased slightly and then plateaued while the PL bundle strain dropped significantly (Fig. 7A) maintaining a similar trend as seen from the in-vivo study by Beynnon et al. [43]. Additionally, Amis and Dawkins (1991) [57] and Hollis et al. (1991) [58] have shown that the AM bundle is tight in extension and stays tight with increasing flexion, while the PL bundle becomes slack with the increase in flexion angles under physiological loading conditions involving flexion; the computational strain trends clearly show this behaviour. However, the computational strains start at higher values due to the associated pre-strains of 6% and 10% at extension.

### Jump-landing simulation

Computational models have been previously developed to understand ACL injury mechanics, specifically during a jump landing event [24, 46]. A common theme among some of these previous models is that the muscle forces have been pre-set values, without consideration for the appropriate model kinematics and dynamics. Therefore, they have not adequately represented the physiological loading conditions during a jump landing event. Recently, rigid body modelling has been used to calculate time-varying muscle forces as boundary condition inputs to FE models. Navacchia et al. [26] developed multiple knee FE models to simulate drop landing and optimize ligament properties to match experimental data collected in an impact simulator. They quantified how external knee loads affect tibiofemoral contact location and forces, and ACL force. Ueno et al. [27] investigated ACL loading during a landing using OpenSIM and FE model to find the relationship between ACL loading and biomechanical factors of individual landing strategies. However, while the study of Ueno et al. [27] investigated the frontal plane biomechanics, the current study chose to investigate sagittal plane kinetic and kinematic factors on the resulting ACL strain. Further, the uniqueness of the current study is in development of an empirical model that quantifies the effect of kinematic parameters on ACL strain.

An extensive study on ACL behaviour during single-leg jump landing was carried out by Bakker et al. [23] examining in-vitro jump landing simulations of seven different participant profiles on five cadaver specimens. Peak ACL strains from the current study were compared with the experimental results from Bakker et al. [23]. The peak ACL strain and the strain trend of the model agreed well with the experimental results, demonstrating the efficacy of the model’s response under dynamic conditions. However, there are some differences between the current study and that of Bakker et al.. Since Bakker et al. used several cadaver tissues, the empirical model to predict the ACL strain had a significant variability represented by a “knee anatomic constant”. The constant, specific to each knee, represented the intrinsic factors (geometry, mechanical properties, etc.) of the knee. This intrinsic factor explained more than 80% of the ACL strain in that study. The current study developed an empirical model without this constant because for each of the ten simulations, same model was used. Further, due to the experimental limitations, the empirical model in the current study considers the contribution of knee and ankle flexion angles in addition to hip and trunk flexion angles found in Bakker et al. [23] study. Further, Bakker et al. [23] did not have ankle moments applied to the cadaver knees during the simulations; however, the current research did apply the time varying ankle moments during landing. Hence, the FE simulations in the current study is considered to more closely represent knee loading than the Bakker et al. approach [23]. It was observed that the application of ankle moment resulted in a 35% reduction in average peak ACL strain compared to when no ankle moment was applied (Fig. 8). Even at low flexion angles, it has been shown in the study by Creswell et al. (1995) [59] that the soleus muscle contributes more to ankle plantar flexion than the gastrocnemius [56]. Hence, as the knee flexes, it is reasonable to assume that the soleus is a primary contributor to the ankle flexion moment. At lower flexion angles, the gastrocnemius muscle is an antagonist of ACL. With an external stimulation of the gastrocnemius muscle group, the study found that the ACL strain increased at lower flexion angles, up to 15 degrees. Elias et al. (2003) [60] reported that the soleus muscle produces a moment at the ankle which rotates the proximal tibia and causing it to move posteriorly. This clearly explains the reduction in ACL strain due to the moment caused by the soleus muscle at the ankle. Mokhtarzadeh et al. (2013) [61] confirmed the findings of Elias et al., [60] demonstrating the antagonistic-agonistic roles of gastrocnemius and soleus respectively.

The sagittal plane parameters involved in the multivariate regression equation (maximum knee flexion, ankle flexion, hip flexion and trunk flexion at maximum GRF) implies a landing scenario consistent with the findings of previous investigations on ACL injury. For instance, Bakker et al. [23] concluded that erect postures (i.e., landing with lower hip and trunk flexion angles) were detrimental to the ACL, which is consistent with the current regression model. Several researchers have investigated this scenario and found similar conclusions. With the study of drop landings of forty participants, Blackburn and Padua [62] showed that actively increasing the trunk flexion angle during landing drives a concomitant increase of hip and knee flexion angles, and thereby associated with lower ACL injury risk. Further, a follow-up study by the same authors [6] revealed lesser ground reaction forces and quadriceps activity and hence resulting in lower ACL forces due to flexion of the trunk during landing. Hence, landing with a flexed trunk, hip and knee angles is favourable to the ACL.

Hashemi et al. (2011) [5] found that in a jump landing activity, co-flexion of the hip and knee joints naturally occurs during landing due to the ground reaction force. They hypothesized that impaired co-activation of quadriceps and hamstrings could result in delayed hip flexion, causing anterior tibial translation which is primarily resisted by the ACL, possibly leading to an injury. Laughlin et al. (2011) [63] stressed the importance of ‘soft’ landing technique (maximizing knee flexion), specifically the role of hamstring forces in increasing posterior shear forces on the tibia resulting in reduced peak ACL forces, post-landing. The same study found that the reduction in peak force was mainly due to landing at higher knee flexion angles, in combination with higher hip flexion angles. The presence of the hip and knee flexion terms in the regression equation from the current study not only supports these theories proposed by Hashemi et al. [5] and Laughlin et al. [63] but also provides quantification of the effects.

Ultimately, a major contribution of this study is the presentation of an empirical model that can be used to predict ACL strain during drop landings, without the need for taking anatomical factors into account. This could be useful in athletic training when proper landing strategies are taught by varying the sagittal plane parameters. For example, the model could approximate the difference in ACL strain when an athlete increases their knee flexion by 10 degrees but decreases their trunk flexion by 5 degrees during a drop landing.

### Limitations

The computational finite element (FE) study presented here acknowledges certain limitations, which were carefully considered during the simulation process. One major concern addressed was ensuring that the FE simulation adhered to the law of conservation of energy to maintain accuracy and validity.

To accurately represent the ligament attachment sites, digitization techniques were employed. However, limitations arose when determining the ligament slack-taut transitions and pre-strain values, which were based on published population average values rather than individual cadaver specimen data. The FE model's accuracy could be improved by obtaining and assigning ligament properties from specific cadaver specimens. Despite these limitations, the kinematic responses, including ACL and meniscal strain results, were found to be reasonable and valid. The study focused solely on modeling the superficial MCL, omitting its interaction with the medial meniscus. Nevertheless, this omission is unlikely to significantly impact sagittal plane knee mechanics.

Another limitation lies in the anisotropic behavior of soft tissues like cartilage and menisci, which were modeled as isotropic, linearly elastic materials. Consequently, the values of tibiofemoral and patellofemoral contact forces and cartilage stresses may not be fully accurate and require further investigation. However, the overall ACL strain results were determined to be less sensitive to cartilage properties based on preliminary pilot studies. One significant drawback is that ligaments were modeled as 1D spring elements rather than 3D structures, preventing a detailed strain distribution analysis within the ligaments.

Only a sagittal plane mechanism was investigated in this research study due to the association with drop landings. However, it is well known that ACL injury mechanisms are also influenced by frontal plane biomechanics [2, 11]. Future research with this modeling approach should consider dominate frontal plane activities such as plant-and-cut.

The FE knee model used in the study was originally developed in-house over a number of years specific for this project. Fortunately, there have been several more advanced and open source knee models now available through Open Knee library (Chokhandre et al. (2023) [64]. Future work could look to replicate this study using these available models, where the effects of geometric parameters as well as extrinsic factors could be studied concurrently.

Notwithstanding these limitations, the computational approach presented in this study remains valuable for predicting injury risk in dynamic loading scenarios. Although further refinements and investigations could enhance accuracy, the study's findings can contribute significantly to understanding injury mechanisms and potential preventative measures.

## Conclusions

A computational model was developed and comprehensively validated to simulate the dynamic conditions of a single-leg jump landing event and to isolate the extrinsic factors affecting ACL strain. Simulations of single-leg jump landings produced reasonable predictions that agreed well with previously reported experimental results. The empirical model provides valuable insight into ACL mechanics demonstrating the influence of sagittal plane parameters, the role of the soleus muscle on knee kinematics. The model can be used to predict ACL strain without any invasive instrumentation. Such a model can be used to train athletes to reduce the risk of ACL injury.

### Supplementary Information

**Supplementary Material 1.**

## Data Availability

The datasets used and/or analysed during the current study are available from the corresponding author on reasonable request.
