# Supplementary material for: Computational study of extrinsic factors affecting ACL strain during single-leg jump landing
Source: BMC Musculoskelet Disord. 2024 Apr 23;25:318. doi: 10.1186/s12891-024-07372-7 (PMC11036765; doi:10.1186/s12891-024-07372-7)
Supplement: Supplementary file 1 — Supplementary Material 1. [file 12891_2024_7372_MOESM1_ESM.docx]

Supplemental Data

Table A1. Summary of the material properties assigned to various structures in the knee model

| Anatomical region | Definition in Abaqus | | Young’s modulus (MPa) | Poisson’s ratio | Reference study |
| --- | --- | --- | --- | --- | --- |
| Femur | | Rigid | 8000 | 0.3 | Haut Donahue, Hull, Rashid, & Jacobs (2002) |
| Tibia | |  |  |  |  |
| Patella | |  |  |  |  |
| Fibula | |  |  |  |  |
| Femoral cartilage | | Deformable | 20 | 0.45 | Oloyede, Flachsmann, & Broom (1992) |
| Lateral tibial cartilage | |  |  |  |  |
| Medial tibial cartilage | |  |  |  |  |
| Lateral meniscus | | Deformable | 59 | 0.49 | LeRoux & Setton (2002) |
| Medial meniscus | |  |  |  |  |

Table A2: Properties assigned to the major knee ligaments in the knee FE model

| Ligament | Ligament bundle | Stiffness parameter ($\boldsymbol{k}$) | Reference strain, $\boldsymbol{\varepsilon}_{\boldsymbol{r}}$ (at extension) | Reference study |
| --- | --- | --- | --- | --- |
| ACL | Anteromedial (AM) | NA | 0.06 | Chandrashekar (2005); Blankevoort et al. (1991) |
|  | Posterolateral (PL) |  | 0.10 |  |
| PCL | Posteromedial (PM) | 9000 | -0.15 | Blankevoort & Huiskes (1996) |
|  | Anterolateral (AL) | 9000 | -0.03 |  |
| Superficial MCL | Anterior (aMCL) | 2750 | 0.04 | Blankevoort et al. (1991) |
|  | Middle (mMCL) | 2750 | 0.04 |  |
|  | Posterior (aMCL) | 2750 | 0.03 |  |
| LCL | Anterior (aMCL) | 2000 | -0.25 | Blankevoort et al. (1991) |
|  | Middle (aMCL) | 2000 | -0.05 |  |
|  | Posterior (aMCL) | 2000 | 0.08 |  |

Table A3: Participant information for jump landing trials, adapted from Bakker et. al(2016)

| Participant | Sex | Body mass (kg) | Limb length (mm) | |
| --- | --- | --- | --- | --- |
|  |  |  | Femur | Tibia |
| P1 | F | 59.0 | 399 | 368 |
| P2 | F | 61.0 | 422 | 404 |
| P3 | M | 72.0 | 429 | 396 |
| P4 | M | 79.0 | 422 | 391 |
| P5 | F | 57.5 | 411 | 381 |
| P6 | F | 67.5 | 411 | 381 |
| P7 | F | 66.0 | 437 | 404 |
| P8 | M | 72.5 | 442 | 427 |
| P9 | M | 74.0 | 422 | 391 |
| P10 | M | 65.0 | 404 | 381 |

*Figure A4: A summary of mesh quality of the soft tissues*

Table A5: Bivariate analysis correlating various sagittal plane parameters obtained from OpenSIM with the corresponding ACL strain obtained from FE simulations.

| Parameter type | Parameters | Normalized peak ACL strain | | |
| --- | --- | --- | --- | --- |
|  |  | Pearson correlation | p-value | |
|  | Body weight | 0.471 | | 0.17 |
|  | GRF | 0.352 | | 0.318 |
| Kinematics (deg) | Max. ankle flexion angle | 0.221 | | 0.541 |
|  | Max. knee flexion angle | -0.18 | | 0.620 |
|  | Max. hip flexion angle | -0.169 | | 0.641 |
|  | Max. trunk flexion angle | 0.01 | | 0.978 |
|  | Ankle flexion angle at max. GRF | -0.09 | | 0.804 |
|  | Knee flexion angle at max. GRF | -0.623 | | 0.054 |
|  | Hip flexion angle at max. GRF | -0.169 | | 0.653 |
|  | Trunk flexion angle at max. GRF | -0.006 | | 0.988 |
| Joint moments (N.m) | Max. knee extension moment | 0.062 | | 0.864 |
|  | Max. hip extension moment | -0.337 | | 0.339 |
|  | Max. trunk extension moment | -0.084 | | 0.817 |
|  | Knee extension moment at max. GRF | 0.08 | | 0.826 |
|  | Hip extension moment at max. GRF | -0.606 | | 0.063 |
|  | Trunk extension moment at max. GRF | -0.395 | | 0.259 |
| Muscle forces (N) | Max. quadriceps force | -0.068 | | 0.849 |
|  | Max. hamstrings force | -0.153 | | 0.672 |
|  | Max. gastrocnemius force | 0.009 | | 0.979 |
|  | Quadriceps force at max. GRF | -0.148 | | 0.681 |
|  | Hamstrings force at max. GRF | -0.268 | | 0.454 |
|  | Gastrocnemius force at max. GRF | -0.095 | | 0.791 |
| Range of motion (deg) | Knee angle ROM at max. GRF | -0.037 | | 0.920 |
|  | Hip angle ROM at max. GRF | -0.132 | | 0.717 |
|  | Knee/Hip angle ROM at max. GRF | 0.164 | | 0.650 |
| Joint velocity (deg/s) | Knee joint velocity at max. GRF | -0.001 | | 0.995 |
|  | Hip joint velocity at max. GRF | -0.063 | | 0.863 |
